# CONTEXTS SURROUNDING PSYCHOLOGICAL MISTREATMENT AND NEGLECT BY CAREGIVERS TO PEOPLE WITH DEMENTIA

**DOI:** 10.1093/geroni/igz038.2004

**Published:** 2019-11-08

**Authors:** Kylie Meyer, Kyungmi Lee, Maria Yefimova, Carolyn Pickering

**Affiliations:** 1 University of Texas Health Science Center San Antonio, San Antonio, Texas, United States; 2 UT Health Sciences at San Antonio, San Antonio, Texas, United States; 3 Stanford Healthcare, Palo Alto, California, United States

## Abstract

This presentation examines the individual experiences of caregivers using psychological mistreatment and neglect. When a caregiver indicated psychological mistreatment (e.g., yelling) or neglect (e.g., skipping necessary care) occurred on their daily diary, they were asked what they were doing and how they felt when the event occurred. Text responses were analyzed using content analysis. When psychological mistreatment occurred, there was often a triggering event. For example, 43.8% of caregivers reported they were responding to a behavioral symptom, and 28% indicated something inconvenient occurred. Caregivers were mostly frustrated/angry (68.8%) and annoyed (21.9%) when they used psychological mistreatment. When the caregiver neglected the recipient, 43.5% of caregivers reported the recipient refused to receive care and 30% reported prioritizing other care activities. In cases of neglect, caregivers were frustrated/angry (39.1%) and worried/anxious (30.4%). Findings indicate psychological mistreatment and neglect occur in unique contexts; prevention of these behaviors likely will require distinct intervention strategies.

